# The burden of injuries in Iranian children in 2005

**DOI:** 10.1186/1478-7954-8-5

**Published:** 2010-03-31

**Authors:** Mohsen Naghavi, Farshad Pourmalek, Saeid Shahraz, Nahid Jafari, Bahram Delavar, Mohammad Esmail Motlagh

**Affiliations:** 1 Institute for Health Metrics and Evaluation, University of Washington, Seattle, Washington, USA; 2 Harvard University Initiative for Global Health, Cambridge, Massachusetts, USA; 3 Center for Network Development and Health Promotion, Ministry of Health and Medical Education, Tehran, Iran; 4 Tehran University of Medical Sciences, Tehran, Iran

## Abstract

**Background:**

Child injury is recognized as a global health problem. Injuries caused the highest burden of disease among the total population of Iran in 2003. We aimed to estimate the morbidity, mortality, and disease burden caused by child injuries in the 0- to 14-year-old population of Iran in 2005.

**Methods:**

We estimated average age- and sex-specific mortality rates for different types of child injuries from 2001 to 2006 using Iran's death registration data. Incidence rates for nonfatal outcomes of child injuries in 2005 were estimated through a time- and place-limited sample hospital registry study for injuries. We used the World Health Organization's methods for estimation of years of life lost due to premature mortality and years lived with disability in 2005.

**Results:**

Injuries were the most important cause of death in children ages 1 to 14, with 35, 33.4, 24.9, and 22.9 deaths per 100,000 in the 0-14, 1-4, 5-9, and 10-14 age groups respectively. Road transport injuries were responsible for the highest death rate per 100,000 population among all types of injuries in children, with 15.5 for ages 0-14, 16.1 for ages 1-4, 16.3 for ages 5-9, and 13.1 for ages 10-14. Incidence rates of injuries leading to hospitalization were 459, 530, and 439 per 100,000 in the 0-14, 1-4, and 5-14 age groups respectively. Incidence rates of injuries leading to outpatient care were 1,812, 2,390, and 1,650 per 100,000 in the same age groups respectively. Among injury types, falls and burns had the highest hospitalization and outpatient care incidence rates.

**Conclusions:**

Injuries, particularly road transport injuries, were the most important health problem of children in Iran in 2003 and 2005. Strong social policy is needed to ensure child survival.

## Background

Child injuries are recognized as a significant health problem for children and communities at the global level [1]. Globally, injuries are the leading cause of death for 10- to 19-year-old children, with road transport injuries (RTI) the leading cause of death for 15- to 19-year-olds and the second-leading cause in children ages 5 to 14 [1]. Injuries, and most notably RTIs, have been identified as a major health problem in Iran during this decade, when the coverage and quality of national death registration systems improved. The first Iranian national burden of disease study for 2003 indicates injuries had the second-highest disability-adjusted life year (DALY) rate in the 0-4 age group, and RTIs led to the highest rate of DALYs in 5- to 14-year-old children [2]. Hence, we aimed to examine the burden of injuries by their different types among Iranian children for 2005.

## Methods

In this study, the reference year for estimation of child injury DALYs is 2005. For comparison, we used the first Iranian national burden of disease study results for 2003 [2]. The trend of child injury mortality was estimated using death registry data from 2001 to 2006. In order to obtain population rates, we divided children into four age groups: under 1 year; 1-4 years; 5-9 years, and 10-14 years. The rationale for using these age groups is that it is consistent with age groupings used in censuses and other types of demographic and epidemiologic studies. Currently, there is no definitive way for exact delimitation of age groups from 0 to 12 years and up to 16 or 18 years, and that lack of a definitive definition of age groups creates problems for demographic and epidemiologic studies [1]. Injuries due to external causes in this article refer to all events coded as injuries with external causes in Chapter 20 of International Statistical Classification of Diseases and Related Health Problems, Tenth Revision (ICD-10) with three-digit codes [3].

### (A) Mortality

We estimated average age- and sex-specific mortality rates for different types of child injuries from 2001 to 2006 in order to stabilize the age, sex, and cause-specific mortality rates in these small age groups. Numbers of deaths were taken from the death registry of the Ministry of Health and Medical Education (MOHME) [4], and ill-defined and garbage codes of death were corrected [5]. Population figures for 2001 to 2006 were estimated based on published data from the 1996 national census by the Statistical Center of Iran [6], and birth and mortality rates were derived from the 2000 Demographic and Health Survey by MOHME [7]. Iran's death registry started with coverage of one province in 1999 and expanded to cover 29 of the 30 provinces in 2006 [5]. The registry uses data from multiple sources and covers all the country's population except Teheran province. Iran's MOHME national death registry has been found to be a good death registration system, with medical information on the cause of death for almost all of the country's provinces [8] and locality of death recorded as place of residence, not place where death occurred.

### (B) Morbidity

In 2005, we conducted a time- and place-limited sample hospital registry study for nonfatal outcomes of injuries in 12 provinces of Iran. These provinces constituted 33,046,751 people, or 47.8% of the total population of Iran. All hospital inpatient admissions in the 12 provinces were recorded in four separate weeks (first weeks of the third month of each season), covering 7.7% of the year for inpatient data. Outpatient and emergency ward admissions of all hospitals and outpatient care services in all medical facilities in the 12 provinces were recorded in four separate days, one in each of the four selected weeks, covering 1% of the year for outpatient data. Only trivial injuries managed at home without using health care services were not included [5]. This time- and place-limited sampling was designed so that the results are generalizable to the whole country in 2005.

### (C) Burden of injuries

Mortality data from the death registry system in 2005 was used to estimate years of life lost due to premature mortality (YLL). Years of life lived with disability (YLD) was estimated using inpatient and outpatient data. Disability-adjusted life years (DALYs) are the sum of YLL and YLD. We used standard methods for national burden of disease studies to estimate the above-mentioned measures of disease burden [9].

### (D) Comparison with other studies

The Global Burden of Disease study provides burden estimates for diseases and injuries by region and country for 2002 and 2004 [10,11]. We adopted injury definitions and methods used by the World Health Organization (WHO) for our estimations and compare our results with WHO's estimates for Iran, subregion B of the Eastern Mediterranean Region (EMR-B) in which Iran resides, and subregion D (EMR-D).

## Results

### (A) Trend of changes in fraction of deaths due to injuries

**Figure 1:**
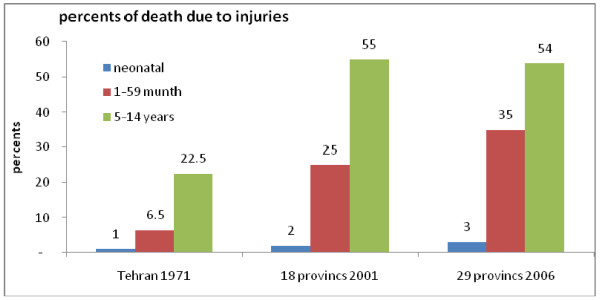
**Proportions of deaths due to injuries in age groups of children in the city of Tehran in 1971 **[12], **18 provinces of Iran in 2001, and 29 provinces of Iran in 2006 **[4].

There is only one published study with cause and age patterns of mortality in Iran before 1990, a study of death patterns in the city of Tehran in 1971 [12]. We used this study to describe mortality trends in Iran because it is a unique study of mortality in Iran before 1990. Figure 1 shows the proportions of deaths due to injuries among children in Tehran in 1971; in 18 provinces of Iran in 2001; and in 29 provinces in 2006 [4,12]. Child injury deaths in Tehran in 1971 are not completely comparable with child injury deaths in 18 and 29 provinces of Iran in 2001 and 2006, respectively, because of differences in population and area covered. However, the 1971 cause-specific data for the city of Tehran is the only data available during the 1970s. Figure 1 indicates a rise in proportion of child deaths due to external causes between 1971 and 2006.

### (B) Pattern of deaths due to all causes and due to injuries,2001 to 2006

In order to derive statistically stable, cause-specific mortality rates, and specifically deaths due to injuries in children, we analyzed the death registry data for 2001 to 2006 and calculated the mean mortality rate for these six years. This analysis indicates an injuries death rate of 35 per 100,000 for the 0-14 age group, constituting 19% of total deaths for this age group, the second-leading cause of death. For the under age 1 population, injuries were the fourth-leading cause of death, with a death rate of 79 per 100,000, or 4% of total deaths for this group. In the 1-4 age group, the injuries death rate was 33 per 100,000, or 42% of total deaths, making injuries the leading cause of death. Injuries were also the leading cause of death for 5-9 and 10-14 age groups, with death rates of 25 per 100,000 and 23 per 100,000, respectively.

Table 1 shows the cause-specific mortality rates for external causes (i.e., injuries) and indicates transport injuries are the leading cause of child death due to injuries. RTIs were responsible for the highest death rate among all child injuries, with 15.5, 16.1, 16.3, and 13.1 deaths per 100,000 in the 0-14, 1-4, 5-9, and 10-14 age groups respectively. This cause-specific mortality rate decreased with an increase in age. After transport injuries, other major causes of death due to external causes in children differ by age. Under 15 years old, deaths due to external causes in Iran from 2001 to 2006 were 35.5 per 100,000 [4]. The above estimates were calculated without taking into consideration deaths due to the Bam earthquake in southeast Iran in 2003 that claimed 8,800 lives among children aged 0-14 [13]. If the child deaths from the Bam earthquake are added, under-15 deaths due to external causes in Iran from 2001 to 2006 amount to 44,300, or an average of 7,000 to 8,000 deaths per year. The most important external cause has been transport injuries, with 18,800 deaths or an average of 3,000 to 4,000 deaths each year. During 2001 to 2006, 3,200 children died of burns, 1,550 died of falls, 1,300 died of obstruction of airways with solid and semi-solid material, 1,200 died of poisoning, and 1,200 children died of intentional injuries in Iran.

**Table 1:** Cause-specific mortality rates per 100,000 for injuries in all ages and both sexes in Iran, mean value for 2001 to 2006, by type of external causes [Source: 4]

Cause-specific mortality rates per 100,000 in 0-14 years and both sexes in Iran, mean value for 2001-6	ICD-10 Code*	under 1 year	1-4 years	5-9 years	10-14 years
Transport injuries	V00-V99	25.3	16.1	16.3	13.1

Other accidental threats to breathing	W75-W84	13.4	0.8	0.2	0.2

Burn	X00-X19	8.5	4.3	1.4	1.8

Exposure to other specified and unspecified factors	W85-W99, X58	7.7	2.1	1.4	1.2

Accidental poisoning by and exposure to noxious substances	X40-X49	6.8	1.2	0.5	0.5

Falls	W00-W19	4.6	1.9	1	0.7

Accidental drowning and submersion	W65-W74	4.3	4.2	1.8	1.9

Complications of medical and surgical care	Y40-Y84	2.3	0.2	0.1	0.1

Exposure to inanimate mechanical forces	W20-W49	1.9	1	0.8	0.6

Exposure to forces of nature	X30-X39	1.7	0.6	0.7	0.6

Contact with venomous animals and plants	X20-X29	1.5	0.6	0.7	0.5

Intentional injuries	X60-Y36	1.1	0.3	0.3	1.9

Overexertion, privation and travel	X50-X57	0.3	0.04	0.01	0.00

Exposure to animate mechanical forces	W50-W64	0.2	0.1	0.04	0.03

* ICD-10: International Statistical Classification of Diseases and Related Health Problems, Tenth Revision [3]

### (C) Incidence and pattern of fatal and nonfatal injuries in 2005

**Figure 2:**
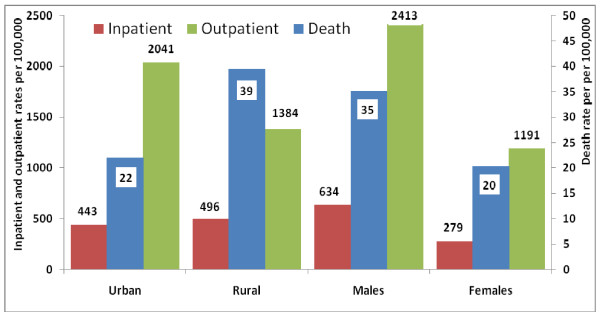
**Rates of outpatient, inpatient, and mortality due to all injuries in 0- to 14-year-old children in Iran in 2005 by sex and urban or rural place of residence**.

**Figure 3:**
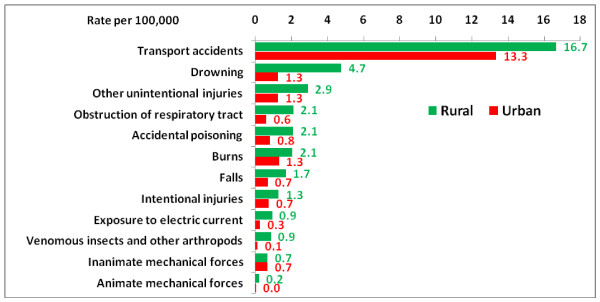
**Injuries mortality rates in the 0-14 age group in Iran 2005 by urban or rural place of residence**.

Incidence rates of injuries leading to hospitalization were 459, 444, 530, and 439 per 100,000 in the 0-14, under1, 1-4, and 5-14 age groups respectively. Incidence rates of injuries leading to outpatient care were 1,812, 1,644, 2,390, and 1,650 per 100,000 in the same age groups. Figure 2 illustrates the large difference in child death rates due to unintentional and intentional injuries between rural and urban areas in 2005, with death rates in rural areas being higher. Although less notable in magnitude, there was a similar difference for inpatient rates. Outpatient rates in urban areas were higher than in rural areas. Figure 3 shows the injury mortality rates in children (0-14 years) by urban or rural place of residence. Mortality rates for all external causes were higher in rural areas than in urban areas, except those for injuries due to inanimate mechanical forces. Boys had higher mortality rates from external causes relative to girls, with the exception of insect bites and venomous arthropod stings.

Table 2 illustrates the incidence rate for injuries leading to hospitalization or outpatient care in Iranian children ages 0 to 14 in 2005 by age, type of care, and cause. Incidence rates for nonfatal outcomes of injuries due to falls were higher than those for transport injuries in almost all of the age groups. In almost all cases, the incidence rates of nonfatal injuries were higher in boys than in girls.

**Table 2:** Incidence rates of injuries leading to hospitalization or outpatient care per 100,000 in the 0-14 age group in Iran in 2005 by age, type of care, and type of cause

Type of care	Hospitalization			Outpatient Care		
Age group (year)	0	1-4	5-14	0	1-4	5-14

Unintentional and intentional injuries (all)	444	530	439	1644	2390	1650

Unintentional injuries (all)	436	525	423	1627	2377	1599

Falls	149	182	155	565	860	480

Burns	74	62	13	415	371	99

Exposure to inanimate mechanical forces	45	74	59	266	518	382

Transport injuries	67	101	160	186	200	380

Accidental poisoning by and exposure to other gases and vapors	74	74	10	83	148	43

Exposure to animate mechanical forces	5	2	9	50	55	105

Intentional injuries (all)	7	5	16	17	13	50

Exposure to electric current	2	1	1	17	4	8

Obstruction of respiratory tract	7	12	1	17	55	6

Contact with venomous insects and other arthropods	12	14	13	17	89	72

Other nonintentional injuries	2	4	5	17	55	21

Drowning	0	0	0	0	25	8

### (D) Burden of fatal and nonfatal outcomes of injuries

Table 3 shows the burden of injuries in Iranian children ages 0 to 14 in 2005 in terms of YLL, YLD, and DALYs. For different types of injuries, DALY rates for 0- to 14-year-old children in 2005 were higher in rural areas than in urban areas, except for falls and inanimate mechanical forces. Transport injuries had the highest DALY and YLL rates, and falls had the highest YLD rates. All DALY rates were higher for boys compared to girls (except unintentional poisoning) and for rural areas compared to urban areas, with some exceptions such as falls and exposure to inanimate mechanical forces.

**Table 3:** Burden of injuries in 0- to 14-year-old children of Iran in 2005

Type of injury	**YLL**^1^	**YLD**^2^	**DALYs**^3^
Unintentional and intentional injuries (all)	203612	231524	435136

Unintentional injuries (all)	196532	228101	424633

Transport injuries	106585	58577	165162

Falls	7453	84647	92099

Exposure to inanimate mechanical forces	4894	46393	51287

Accidental poisoning by and exposure to other gases and vapors	8696	16175	24871

Drowning	17825	95	17920

Burns	11311	6212	17522

Other unintentional injuries	13044	3231	16275

Intentional injuries (all)	7080	3423	10503

Obstruction of respiratory tract	7766	2309	10074

Contact with venomous insects and other arthropods	2734	4499	7233

Exposure to animate mechanical forces	541	5439	5980

Exposure to electric current	3555	773	4328

1: YLL = Years of life lost due to premature mortality; 2: YLD: Years of life lived with disability; 3: DALYs: Disability-adjusted life years

### (E) Comparison with other studies

We compared estimates of DALYs due to injuries in the 0-14-year-old population from the current study for Iran in 2005 with three other studies: GBD estimates for WHO-defined world subregions in 2002 [10] and 2004 [11]; and the first Iranian national burden of disease (NBD) study in 2003 [2]. Iran resides in the EMR-B subregion (low adult and child mortality countries of the Eastern Mediterranean region). Comparisons indicated the rate of DALYs due to injuries in children ages 0-4 in Iran in 2003 was higher than those DALY rates in the EMR-B and EMR-D subregions and all 15 WHO-defined world subregions in 2002 except the AFR-D subregion (African countries with high child and adult mortality). For 5- to 14-year-old children, the DALY rate of injuries in Iran in 2003 was higher than in EMR-D countries in 2002. The proportion of DALYs due to injuries compared to all-cause DALYs in the 0-4 age group in Iran in 2003 (14.3%) was higher than the similar proportion for each and all 15 world subregions in 2002 - 11.1% for EUR-C (European countries with low child mortality and high adult mortality) and 10.4% for EMR-B subregions. Iran ranked first in this metric for 0- to 4-year-olds and ranked fourth for 5- to 14-year-old children (34.6%) after EUR-C (38.9%), EMR-B (37.9%), and WPR-B (Western Pacific subregion with low child and adult mortality) (36.6%). Iran's DALYs rate for injuries in children aged 0-4 in 2005 was lower than the average of the same metric for world subregions in 2004 and higher than the EMR-B subregion in 2004. Table 4 demonstrates the DALYs for specific types of injuries in the children of Iran in 2005 and EMR-B and EMR-D subregions in 2004. Our findings in the current study show that 27% and 44% of the burden of injuries in Iran in 2005 was due to RTIs in the 0-4 and 5-14 age groups respectively, whereas in the EMR-B subregion in 2004, these fractions were 42% and 34% respectively. In the Iran NBD study in 2003 [2], 14% and 35% of the total burden in the 0-4 and 5-14 age groups were from injuries, but in EMR-B subregion in 2004, these fractions were 16% and 14%. Injuries led to the highest DALYs in Iran in 2003, with 38% due to RTIs [2].

**Table 4:** Disability-adjusted life years per 100,000 population for specific injuries in 0-4 and 5-14 age groups in Iran in 2005 and EMR-B* and EMR-D* in 2004 [11]

Age group	0-4 years			5-14 years		
**Cause**	EMR-B*	EMR-D*	**Iran**	EMR-B*	EMR-D*	**Iran**

All Injuries	2652	4513	2892	2164	3643	1816

Unintentional injuries	2380	3008	2879	2114	3162	1751

Road transport injuries	1110	917	787	736	791	808

Poisoning	52	87	220	51	59	83

Falls	282	379	634	344	523	376

Burns	114	320	213	152	386	37

Drowning	103	214	145	107	253	65

Other unintentional injuries	719	1091	822	723	1149	325

Intentional injuries	272	1505	14	50	481	65

Self-inflicted injuries	129	225	0	21	55	46

Violence	125	308	14	27	92	19

War	9	957	0	1	331	0

Other intentional injuries	8	15	0	0	2	0

* EMR-B: Eastern Mediterranean Region, subregion B; EMR-D: Eastern Mediterranean Region, subregion D

### (F) Detailed description of main types of injuries in children

Our estimates for deaths, hospitalization, and outpatient care due to specific injuries in children in 2005 are below.

### (F1) Transport Injuries

Pedestrians comprised 33% of deaths, 42% of hospitalizations, and 41% of outpatient care stemming from transport injuries in children of Iran. Of these hospitalized child pedestrians, 53% were run over within the city streets, and 26% were run over beside city streets (on sidewalks). Among child deaths due to transport injuries, 43% were passengers of cars and pickup trucks. In addition, 19% of hospitalizations and 11% of outpatient care resulting from transport injuries occurred in children as passengers of cars and pickups. Among child passengers of cars and pickups who died or were injured from transport injuries, 34% died in front-to-front collisions and 33% in vehicle rollovers. Twelve percent of deaths, 36% of hospitalizations, and 12% of outpatient care stemming from transport injuries in children occurred in those riding motorbikes (sitting behind the adult rider), of which 43% of events occurred because children fell or were thrown from a motorcycle (without collisions) and 42% occurred during collisions.

### (F2) Falls

The most frequent places in which children died due to falls were in transit (including sidewalks, streets, and the areas around them) (33%), home (33%), and school (17%). The most frequent places in which children suffered nonfatal injuries from falls leading to hospitalization were home (60%), transit places (11%), and schools (10%). Stairs and falling on the same level were the most frequent causes of falls in home and in school. Within the category of falls, transit, falls on the same level, and falls from or into holes and wells were the most frequent types. In play places, falls involving playground equipment and falls on the same level were most frequent. In general, falls from stairs and falls into wells caused the highest proportions of deaths. Falls from stairs, falls on the same level, and falls from a balcony or roof resulted in the highest hospitalization rates.

### (F3) Burns

Burns leading to death were most frequently due to fires caused by gasoline and petrol (72%). Hot liquids were the most frequent burns leading to hospitalization (38%). Outpatient care for burns was most frequently due to hot liquids and hot substances (65%). Burns due to fire from gasoline, petrol, and wood were more frequent in rural areas (54%), and burns with hot liquids were more frequent in urban areas (47%). Child burns occurred more than 60% of the time while playing, with less than 10% related to cooking and about 10% occurring during rest.

### (F4) Injuries due to inanimate mechanical forces

The most frequent types of child exposure to inanimate mechanical forces were sharp and cutting objects (64%); entrapment of a child's body in mechanical instruments and tools (16%); being struck by nonsharp mechanical tools such as a sledge or spade (7%); unintentional gunshot (2%); and the falling of heavy objects on the child (1%). Sixty-five percent of exposure to inanimate mechanical forces occurred in the home, 13% in transit places, 7% in the workplace, 6% in schools, and 6% in play and recreation places. Fifty-eight percent of child exposure to inanimate mechanical forces occurred during playing, 14% during work, 13% during transit, and 3% during rest.

### (F5) Exposure to animate mechanical forces

Injuries due to animate mechanical forces were due to animal bites (32%); being unintentionally stricken by part of another person's body (hand, foot, head) (31%); being crushed, pushed, or stepped on by a crowd (9%); and being beaten or struck by animals' kicks or horns (8%).

### (F6) Poisoning

Poisoning occurred mostly with medicines (41%); petrol (17%); insecticides, rodenticides, pesticides (9%); opioids (8%); carbon monoxide gas (4%); and poisonous plants and mushrooms (4%). Overall, inadvertent consumption of poison by children occurred during play in 26% of the cases; consumption of poison provided unintentionally by another person in 9% of cases; unintentional mixing of poison with food or food ingredients in 6% of cases; and poisoning during work in 3% of cases.

### (F7) Drowning

The most common places for child drowning were traditional water reservoirs, water ponds and puddles (24%), small and big water pools (20%), sea (16%), and river (12%).

### (F8) Obstruction of airways with solids and semi-solidmaterial

Obstruction of airways occurred with foreign solid material in 52% of cases, with food (28%), and suffocation in bed due to position of child's head under pillow or mother's body (5%).

### (F9) Stings by insects and reptiles

Scorpions caused 65% of stings (and more than 70% of hospitalizations and 100% of deaths). Bees caused 12% (and more than 50% of outpatient care cases), snakes 9%, and tarantulas 2%. About 76% occurred at home, 11% in streets and roads, and 7% in schools or playing places.

## Discussion

Injuries were the most common cause of death in 1- to 14-year-old children and the second highest cause of death in the 0-14 age group in Iran. RTIs were responsible for the highest death rate among all child injuries. Among injury types, falls and burns had the highest hospitalization and outpatient care incidence rates.

The proportion of child deaths due to injuries, compared to all child deaths, increased from 1971 to 2001-2006 (Figure 1). This increase results from a combination of a true rise in injury deaths and a decline in other competing causes of death. The reason the mortality rate from injuries decreases as children age is likely due to their improved ability to avoid or escape dangerous settings but is not due to an increase in the provision of safe environments for children.

Limited access to hospital services and more unsafe environments in rural areas may explain the higher child death rates, inpatient rates, and outpatient rates due to injuries in rural areas as compared to urban areas in 2005 (Figure 3). Our finding of lower use of outpatient services due to injuries in rural areas may be due to a limitation of our study method in which urban emergency health services are captured more than similar services in rural areas. Incidence rates of all external causes were higher in boys compared to girls, except for insect bites and venomous arthropod stings. Higher incidence rates of nonfatal transport injuries in rural areas in 2005 are likely due to higher use of motorcycles for transportation [14] and poor car quality, road safety, driver behavior, and access to health services.

The risk factors that caused transport injuries to be the most important external cause of death in Iran in 2003, according to the first Iranian NBD study [2], have not improved substantially from 2003 to 2005. The risk factors were: the lack of laws mandating use of child seats in cars, a practice that is viewed as a luxury; and unsafe transportation practices, including children riding on the back of motorcycles, three to four family members riding on a motorcycle, children sitting in front seats of cars, and children riding in the back of pickup trucks.

Roudsari et al studied childhood trauma fatality from September 1999 to September 2000 in Iran and concluded that unintentional injuries accounted for 94% (394 cases) of fatal childhood injuries. Motor vehicle crashes (50%), burns (18%), falls (6%), and poisonings (6%) were the most common mechanisms of fatal injuries [15]. Other studies similarly have found that injuries, and specifically RTIs, have had the highest mortality rates in 10 out of the 28 provinces in Iran in 2000 [16]. Wazana performed a critical review of the literature on pedestrian injury. He concluded that child risk factors make a consistent but minor contribution to injuries in comparison with environmental and social risk factors (e.g., traffic volume or visual obstacles and poor supervision or family stress, respectively), and that there is a need to advocate for engineering and legislative interventions [17]. Children who are injured or die as a result of injuries do not have intrinsic traits that expose them to significantly more risk of being injured or dying due to injuries. The adult populations of countries are responsible for providing safe environments for children and should be accountable for ensuring children's rights.

The United Nations Convention on the Rights of the Child indicates that children have the right to survival [18]. This right includes living in a safe environment that keeps children from injury and death from injuries. However, the adequacy and effectiveness of environments to prevent external causes of injury and death in children are not sufficient. Injuries impose a large burden of disability and death on children that can be largely prevented. Child safety legislation and the provision of child-safe environments in Iran are almost negligible.

Under-registration of death counts in death registry systems leads to an underestimation in mortality levels [8,9]. The current study is not an exception. We used time- and place-limited hospital sample registry for collecting hospitalization and outpatient data. Selection of time was designed to capture the events across the four seasons of the year 2005, so that the time-limited sample results can be generalized to the whole year. Selection of place (i.e., choosing the provinces) was done to include provinces with higher, middle, and lower socioeconomic and health development so that the results can be generalized to all provinces. The validity of our generalization of results from time- and place-limited samples depends on the suitability of the design of times and places chosen to represent the year 2005 for all of Iran. Moreover, capturing outpatient care episodes in rural areas is more prone to underestimation than in urban areas.

## Conclusions

As a society, Iran has undergone a demographic and health transition, with a shift in disease burden from communicable diseases and perinatal, maternal, and nutritional conditions to noncommunicable diseases and injuries. Child mortality decreased [19] because of increased immunization coverage, oral rehydration therapy for diarrhea, appropriate treatment of acute respiratory infections, and a decrease in malnutrition. Each of these improvements was made possible by an effective primary health network system and improved socioeconomic development. In contrast to these achievements, no effective health program is operational that addresses noncommunicable diseases or injuries. Health system reform should focus on the most burdensome health problems of the community.

At the current level of population health in Iran, provision and maintenance of child safety from injuries is the first and foremost need to address a child's right to survival. Many homes, streets, schools, and playing places are unsafe for Iranian children. The consequences are the deaths of 7,000 to 8,000 children from injuries every year and 70,000 to 80,000 children hospitalized, many of whom live with the burden of disability for the rest of their lives.

Child safety is not the primary responsibility of any governmental or non-governmental organization. Governmental bodies whose responsibilities include child safety, such as the police, conduct sporadic initiatives to reduce child injury. However, results of formal evaluations of their effectiveness have not been published. Transport injuries kill 3,000 to 4,000 children yearly. Safety measures and regulations regarding the construction of children's educational and playing spaces are seldom mandated by law or enforced. The construction of living places does not consider child safety. Unsafe environments for children are worse in rural areas and city peripheries. Unfortunately, the observed decrease in death and hospitalization with an increase in children's ages is not due to the provision of safer environments. This decrease is due to children's increasing ability to escape environmental hazards. Those who are lucky enough to survive unsafe environments in younger ages tend to reach older ages. Prerequisites for effective child safety include, but are not confined to, accountable, transparent, and high-level political commitment to decrease the burden of injuries in all ages and effective management at middle and peripheral levels of government.

Child survival and safety regarding environmental hazards and injuries should not resemble the survival of the fittest through natural selection of individuals who manage to survive an unsafe environment. Strong policies and political and community will are needed to reverse the trend of fatal and nonfatal outcomes of child injuries. Iran has been successful in protecting children against vaccine-preventable diseases, pneumonia, diarrhea, and malnutrition. Now Iran has to reduce child injuries. We quantified the magnitude of child injury burden and compared it to other causes of death and disability and other countries in order to underline the relative importance of this health and social problem. Good intentions, strong opinions, endless reorganizations, and millions of dollars are not enough to bring about a safe childhood [20]. Strong social and health policy is required.

## Competing interests

The authors declare that they have no competing interests.

## Authors' contributions

MN conceived of and led the study, adapted the methodology, and performed the statistical analysis. MN, FP, SS, NJ, BD, and MEM contributed to study implementation. FP, SS, and NJ contributed to statistical analysis. FP and MN drafted the manuscript, and all authors read and approved the final manuscript.
